# Transcription Factor OsbZIP60-like Regulating *OsP5CS1* Gene and 2-Acetyl-1-pyrroline (2-AP) Biosynthesis in Aromatic Rice

**DOI:** 10.3390/plants13010049

**Published:** 2023-12-22

**Authors:** Gegen Bao, Umair Ashraf, Lin Li, Jingxuan Qiao, Chunling Wang, Yixiong Zheng

**Affiliations:** 1 Guangzhou Key Laboratory for Research and Development of Crop Germplasm Resources, Zhongkai University of Agriculture and Engineering, Guangzhou 510225, China; lilin981001@163.com (L.L.); m13704723105_1@163.com (J.Q.); 2 College of Agriculture and Biology, Zhongkai University of Agriculture and Engineering, Guangzhou 510225, China; gdsscqs@163.com; 3 Department of Botany, Division of Science and Technology, University of Education, Lahore 54770, Pakistan; umairashraf2056@gmail.com; 4 College of Life Science, Huizhou University, Huizhou 516007, China; wangcl@hzu.edu.cn

**Keywords:** fragrant rice, 2-acetyl-1-pyrroline, *OsP5CS1*, OsbZIP60-like

## Abstract

The most important volatile in determining the aroma of fragrant rice is 2-Acetyl-1-pyrroline (2-AP); however, the transcriptional regulation mechanism of 2-AP biosynthesis in fragrant rice is still unclear. In this study, *Osp5cs1* knockout mutant lines and *OsP5CS1* over-expression lines were constructed by the genetic transformation of the Indica rice cultivar, i.e., ‘Zhonghua11′, which knocks out *OsBADH2* to produce fragrance in aromatic rice. The *OsP5CS1* gene was also identified as a key gene in the 2-AP biosynthesis pathway of aromatic rice. The *OsP5CS1* promoter was used as bait, and the OsbZIP60-like transcription factor was screened by yeast one-hybrid assays. The OsbZIP60-like transcription factor specifically bound to the *OsP5CS1* gene. The dual luciferase reporting system found that the OsbZIP60-like transcription factor promoted the transcriptional activation of *OsP5CS1*. Compared with the wild type, *OsP5CS1* gene expression was significantly down-regulated in the *Osbzip60*-like mutant and resulted in a substantial reduction in 2-AP biosynthesis. Moreover, the *OsP5CS1* gene expression was significantly up-regulated in *OsbZIP60*-like over-expressed plants, and the 2-AP concentrations were also increased, whereas the *Osbzip60*-like mutants were found to be sensitive to Zn deficiency. Overall, the OsbZIP60-like transcription factor promoted the 2-AP accumulation. This study provides a theoretical basis for the transcriptional regulation mechanism of 2-AP biosynthesis and explores the function of the OsbZIP transcription factor in fragrant rice.

## 1. Introduction

Fragrant rice is favored by consumers due to its excellent cooking qualities, aroma, and high economic value [1,2]. The cultivation of fragrant rice in our country has been experiencing the phenomenon of “losing fragrance as it is cultivated more”. The rice cannot meet the market demand for high-quality fragrant rice, resulting in long-term reliance on imports. The annual import volume has reached 3 million tons [3]. The development of high-quality germplasm resources and the establishment of high-quality and high-yield cultivation technology system for fragrant rice are two important steps to promote the formation of fragrant rice quality [4,5].

**Figure 1:**
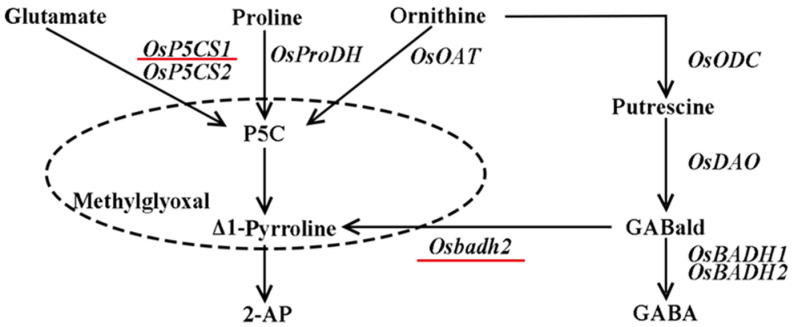
2-AP biosynthetic pathway.

Fragrant rice is a special type of rice where the stems, leaves, panicles, flowers, and grains emit fragrance, apart from the roots [2]. The fragrant aroma of cooked fragrant rice and its rich nutritional content, including various amino acids, have made it highly favored by people in rice-consuming regions around the world [1]. Rice aroma contains about 200 volatile compounds, whereas 2-acetyl-1-pyrroline (2-AP) is considered the most important compound for aroma production in fragrant rice [6,7,8]. There are two biosynthetic pathways for 2-AP: firstly, proline, ornithine and glutamate are catalyzed by proline dehydrogenase (PRODH), ornithine aminotransferase (OAT) and pyrroline-5-carboxylic acid synthase (P5CS), respectively, to form pyrroline-5-carboxylic acid (P5C), which directly reacts with methylglyoxal (MG) to form 2-AP in a non-enzymatic reaction [9], and/or P5C is decarboxylated to form Δ1-Pyrroline catalyzed by pyrroline-5-carboxylate decarboxylase (P5CR), and Δ1-Pyrroline directly forms 2-AP with MG [10,11]. Secondly, ornithine is catalyzed by ornithine decarboxylase (ODC) to form putrescine, and then diamine oxidase (DAO) catalyzes putrescine to form γ-aminobutyraldehyde (GABald). The GABald is converted to γ-aminobutyric acid (GABA) catalyzed by a functional betaine aldehyde dehydrogenase (BADH), which inhibits the biosynthesis of 2-AP in non-aromatic rice. Conversely, GABald cannot be converted to GABA under the action of a nonfunctional betaine aldehyde dehydrogenase (badh), resulting in the accumulation of 2-AP in aromatic rice (Figure 1) [12].

With the establishment of the genetic map of rice, important QTLs and potential candidate genes for explaining the molecular characteristics of fragrance in fragrant rice have been identified [13]. The betaine aldehyde dehydrogenase gene *BADH2*, which was encoded on chromosome 8 of rice, was considered to be directly related to 2-AP biosynthesis, and it is also the focus of research on 2-AP biosynthesis mechanism at present. The *BADH2* gene has multiple mutation types in different fragrant rice cultivars [14]. The most common type of mutation in fragrant rice cultivars was the *BADH2* gene with eight base deletions and three nucleotide polymorphisms in exon 7 [12]. Other types of mutations in fragrant rice cultivars include 7 base deletions in the second exon of the *BADH2* gene, 803 bp deletion between exons 4 and 5, and mutation sites in exons 1, 10, 13, and 14. There were insertions, deletions, and nucleotide polymorphisms in exon 1 and intron 1 regions [4]. These gene mutations produce the enzyme inactive BADH2 protein, which in turn reduces the consumption and content of 2-AP precursor GABald, which in turn promotes the production of 1-pyrroline and ultimately leads to the accumulation of 2-AP in aromatic rice [12]. The *OsBADH2* gene encoded a functional betaine aldehyde dehydrogenase and inhibited 2-AP biosynthesis in rice [9]. The down-regulation of *OsBADH2* gene expression by RNAi interference techniques and TALEN technology resulted in the elevation of 2-AP content in non-aromatic rice [4,14]. The CRISPR/Cas9-mediated editing of the *OsBADH2* gene induced 2-AP production in fragrant rice [15]. Moreover, proline is an important precursor for 2-AP biosynthesis [9]. The P5C synthase encoded by the *OsP5CS* gene has dual functions as glutamate kinase and γ-glutamyl phosphate reductase and plays its role as a rate-limiting enzyme in proline biosynthesis [16]. The rice genome contains two homologous *OsP5CS* genes, i.e., *OsP5CS1* and *OsP5CS2*. *OsP5CS1* is located on chromosome 1 and is often induced by salt, dehydration, and cold stress, whereas the *OsP5CS2* is located on chromosome 5 and is induced by salt and mannitol stress [17,18]. Over-expression of the *OsP5CS1* gene in aromatic rice increased 2-AP content [19,20]. Compared with conventional rice, the expression of the *OsP5CS1* gene in aromatic rice was positively correlated with 2-AP accumulation [21]. There have been many studies on the regulation of *P5CS* expression in different plant tissues in response to different environmental signals, but little is known about the transcriptional regulation of the *P5CS* gene. Cis-acting element analysis of promoters of *OsP5CS1* and *OsP5CS2* genes in rice showed that binding sites of 24 different types of transcription factors were detected in the promoters of the two genes. This includes transcription factor families such as AP2, bZIP, MYB, and NAC [3]). So, determining the transcription factors that bind to the promoter of the *OsP5CS1* gene and how the expression of the *OsP5CS1* gene is regulated to affect the content of 2-AP has not yet been studied.

The long-term non-degradation of aroma in the origin of aromatic rice was closely related to the high zinc (Zn) content in soil [22]. Previously, the foliar application of Zn fertilizer promoted the accumulation of fragrance in fragrant rice. Mo et al. [2] pointed out that exogenous Zn fertilizer application promoted the accumulation of 2-AP in detached panicles of fragrant rice by increasing proline content. Luo et al. [23] found that the foliar application of Zn fertilizer enhanced P5CS enzyme activity, leading to an increased accumulation of 2-AP in fragrant rice. Bao et al. [3] reported that foliar Zn application up-regulated the expression of the *OsP5CS1* gene and promoted the accumulation of 2-AP in aromatic rice.

Zn, as a cofactor in enzymes, transcription factors, and protein–protein interaction domains, plays structural and catalytic roles in many proteins, making it an essential micronutrient for plants [24]. Zinc-binding proteins constitute approximately 10% of the proteome in eukaryotes [25]. Plants rely on a tightly regulated Zn homeostasis network to maintain sufficient intracellular Zn levels and prevent toxicity caused by Zn deficiency or excess accumulation [26]. bZIPs are a large family of transcription factors in plants and contain a bZIP domain that consists of a basic domain and a leucine zipper [27]. These residues were located on adjacent helices, with a basic region consisting of approximately 16 amino acid residues containing a nuclear localization signal, followed by a conserved N-XT-R/K motif that directly bound to DNA [28]. bZIP proteins are the most diverse family in plants and have been identified in whole-genome sequences of various species, including Arabidopsis, rice, maize (*Zea mays* L), sorghum (*Sorghum bicolor* (L.) Moench), grape (*Vitis vinifera* L.), tomato (*Lycopersicon esculentum* Mill.), and apple (*Malus pumila* Mill.) [27]. Seventy-five putative genes encoding bZIP proteins have been identified in Arabidopsis. These bZIP proteins were classified into 10 groups based on sequence domain similarity and biological function [29]. The F-bZIP proteins were the central mediator of Zn deficiency reaction [30,31], whereas the F-bZIP transcription factors AtbZIP19 and AtbZIP23 maintained Zn levels in cells through the N-terminal Cys-his-rich motif and the single-Cys or His-residue binding of Zn^2+^ in *Arabidopsis thaliana* [31,32]. Plants rely on a strict network of Zn homeostasis to maintain Zn levels in the cells, and to avoid damages caused by an excess or deficiency of Zn [26]; however, the regulation of 2-AP by the bZIP transcription factor needs investigations. The targeted editing of the 2-AP biosynthetic gene *OsBADH2* by CRISPR/Cas9 technology and the development of high-quality aromatic rice resources are becoming important. The present study was therefore conducted to develop the *Osp5cs1* knockout mutant and *OsP5CS1* overexpression lines by genetic transformation from the perspective of *OsBADH2* knockout in Indica rice.

## 2. Results

### 2.1. The OsP5CS1 Gene and 2-AP Biosynthesis

**Figure 2:**
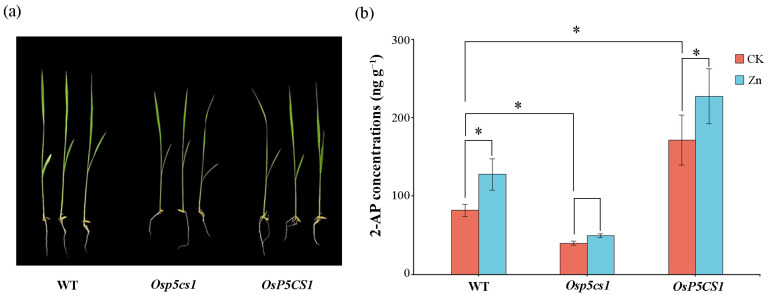
Comparison of 2-AP concentrations in wild type (WT), *Osp5cs1* mutant and *OsP5CS1* over-expressing plants. (**a**) Phenotype of seedlings after the second week of growth (**b**) 2-AP concentrations; CK: controlled water spray treatment; Zn: 30 mg L^−1^ ZnCl_2_ treatment. * means there was a difference at *p* < 0.05 by LSD test.

Compared with wild type (WT), the growth of *Osp5cs1* plants was inhibited, whereas the *OsP5CS1* plants were similar to the WT (Figure 2a). Compared with WT, the 2-AP concentrations in the *Osp5cs1* plants were decreased by 51.11%, and the 2-AP concentrations in the *OsP5CS1* plants were increased by 52.26%. In the WT, the concentrations of 2-AP were increased by 35.98% under Zn treatment compared with CK. In the *OsP5CS1* plants, the 2-AP concentrations were increased by 24.59% under Zn treatment compared with CK (Figure 2b). This result proves that the *OsP5CS1* gene was a key gene in the 2-AP biosynthesis pathway of fragrant rice.

### 2.2. Analysis of Cis-Acting Elements in the OsP5CS1 Promoter

Plant CARE was used to analyze the type, quantity and position of the cis-acting elements of the *OsP5CS1* promoter to predict the function of the promoter. It was found that there are many cis-acting elements with different functions in the promoter. There were multiple cis-acting elements that may bind to bZIP60, such as ABRE, ABRE3a, ABRE4, G-Box, and ERE (Table 1).

### 2.3. OsbZIP60-like Transcription Factor

**Figure 3:**
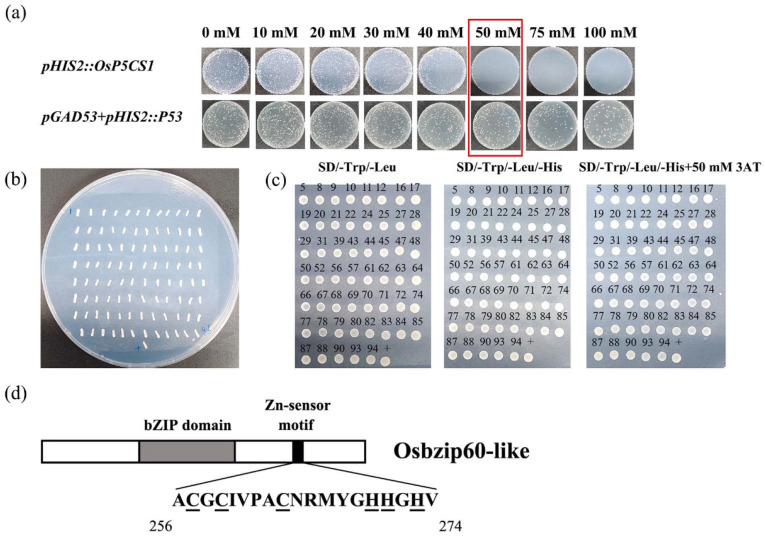
OsbZIP60-like transcription factor screened by yeast single hybrid. (**a**) Background screening of defective plates with different concentrations (0, 10, 20, 30, 40, 50, 75, 100 mM 3AT) of 3AT, (**b**) 96 monoclonal colonies grew on SD-TLH + 50 mM 3AT plates of the screening library, (**c**) Rotational validation of monoclonal; (+) positive control pGAD53m + pHIS2:p53, (**d**) OsbZIP60-like protein structure analysis. C means Cys; H means His.

The *OsP5CS1* gene promoter sequence was ligated into the bait vector and transformed into yeast. No colony growth of transformants on the medium supplemented with 50 mM 3AT was established, indicating that the *HIS3* reporter gene was not activated, so yeast one-hybrid was performed on 50 mM 3AT (Figure 3a). Through the library of rice nuclear genes, 96 positive clones were screened (Figure 3b). After PCR amplification and Seqman and BLAST comparison, the empty and repeated sequences were removed, and the 53 different sequences were obtained. Fifty-three positive clones were inoculated on SD-TLH + 50 mM 3AT-deficient plates, diluted with sterile water, and then placed on SD-TL-, SD-TLH-, and SD-TLH + 50 mM 3AT-deficient plates for rotary verification (Figure 3c). The protein LOC4344086 appeared several times in the sequencing of positive clones and showed strong positive signals. The sequence alignment showed that the fragment sequence was 100% similar to the OsbZIP60-like gene sequence of rice. According to the amino acid structure analysis, the OsbZIP60-like gene sequence was identified as a group F-bZIP transcription factor, in addition to the BRLZ basic domain and leucine-zipping dimer domain typical of the bZIP family, and the amino acid composition was rich in Cys and His residues binding to Zn^2+^ (Figure 3d). The results preliminarily indicate that the OsbZIP60-like transcription factor specifically binds to the *OsP5CS1* promoter.

### 2.4. The OsP5CS1 Required the OsbZIP60-like for Transactivation

**Figure 4:**
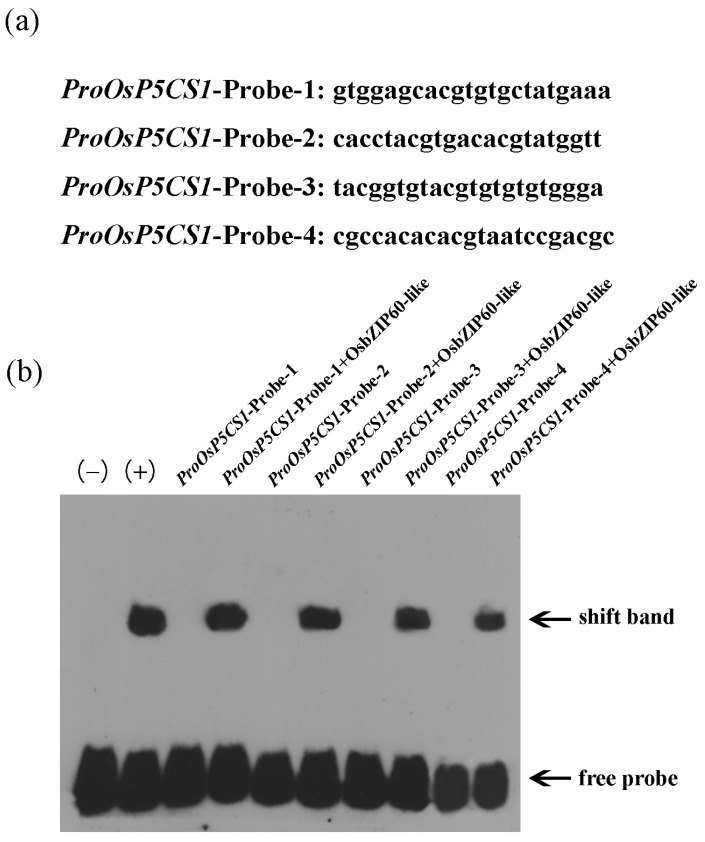
The OsbZIP60-like transcription factor specifically bound to the *OsP5CS1* promoter. (**a**) Oligonucleotide sequences used in EMSA, (**b**) OsbZIP60-like binds to *ProOsP5CS1*-Probe-1, *ProOsP5CS1*-Probe-2, *ProOsP5CS1*-Probe-3 and *ProOsP5CS1*-Probe-4. “−” and “+” denote negative and positive controls, respectively.

Eleven presumed OsbZIP60-like cis-elements were identified in the promoter region of *OsP5CS1* (2000 bp). The *OsP5CS1* promoter was connected to the firefly lucifin reporter (Figure 4a), and the *OsP5CS1* promoter was strongly activated by OsbZIP60-like (Figure 4b).

### 2.5. The OsbZIP60-like Transcription Factor Specifically Bound to the OsP5CS1 Promoter

**Figure 5:**
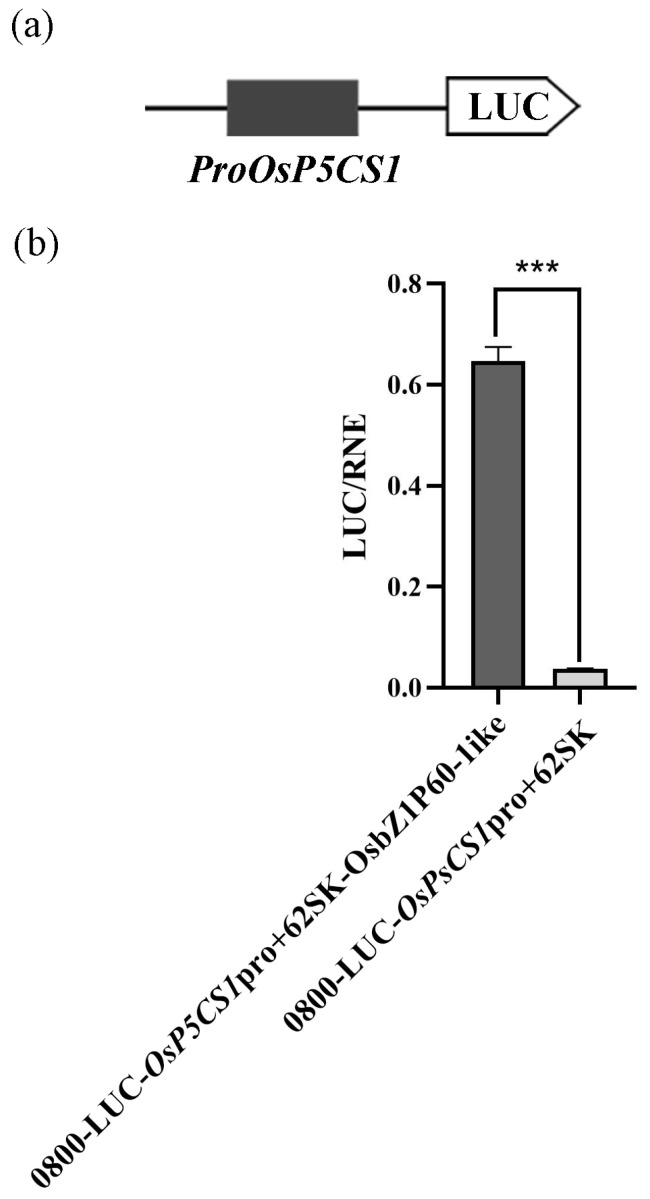
The *OsP5CS1* required the OsbZIP60-like for transactivation. (**a**) The *OsP5CS1* promoter was connected to the firefly lucifin reporter, (**b**) The corresponding relative ratio of LUC/REN was shown on the right. Error bars indicate SE from six replicates. Asterisks indicate significant differences as determined by *t*-test analysis (*p* < 0.01).

The purified OsbZIP60-like protein was incubated with four selected labeled promoter fragments (Figure 5a). Specific DNA complexes were detected in four probes (Figure 5b). The results confirmed that the OsbZIP60-like transcription factor specifically bound to the *OsP5CS1* promoter.

### 2.6. Transcription Factor OsbZIP60-like and 2-AP Biosynthesis

**Figure 6:**
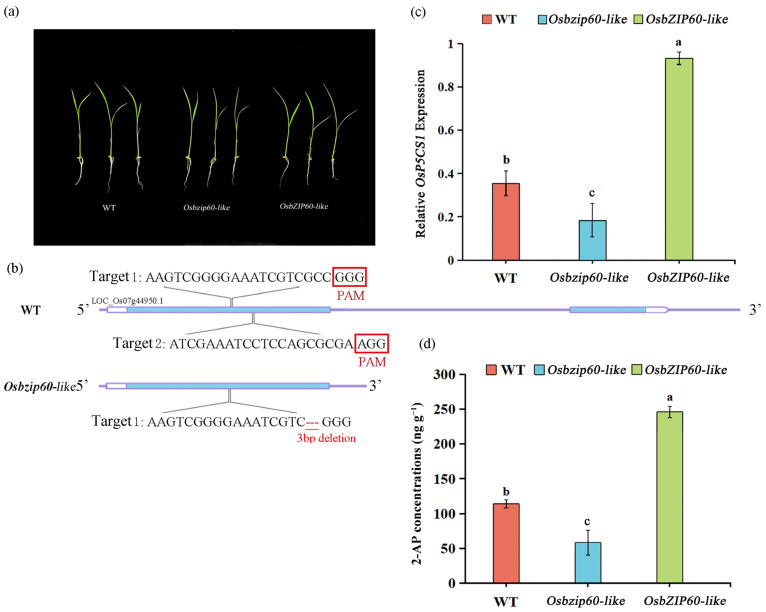
Comparison of 2-AP concentrations in wild type (WT), *OsbZIP60*-like mutant and *OsbZIP60*-like-1-CP complementing lines. (**a**) The growth phenotypes of wild-type (WT), *Osbzip60*-like mutant and *OsbZIP60*-like over-expressed plants at the second-week seedling stage, (**b**) *Osbzip60*-like mutant constructed by CRISPR/Cas9 system, (**c**) wild-type (WT), The expression of *OsP5CS1* gene in *Osbzip60*-like mutants and *OsbZIP60*-like over-expressed plants, (**d**) The determination of 2-AP concentrations in wild type (WT), *OsbZIP60*-like mutants and *OsbZIP60*-like over-expressed plants. Different letters above the bar graphs indicate differences at *p* < 0.05 by the LSD test.

There was no significant difference in the growth of the WT, the *Osbzip60*-like mutants and the *OsbZIP60*-like over-expressing plants (Figure 6a). The 3bp deletion of the target site in the mutant affected the function of the protein (Figure 6b). Compared with the WT, the expression of the *OsP5CS1* gene was down-regulated by 48.65% in the *Osbzip60*-like mutants and up-regulated by 124.32% in *OsbZIP60*-like over-expressing plants (Figure 6c). Compared with the WT, the 2-AP concentrations were decreased by 45.45% in *Osbzip60*-like mutants and increased by 100% in *OsbZIP60*-like over-expressing plants (Figure 6d). Overall, the OsbZIP60-like transcription factor was a positive regulator of 2-AP accumulation.

### 2.7. OsbZIP60-like Mutants Were Sensitive to Zn Deficiency

**Figure 7:**
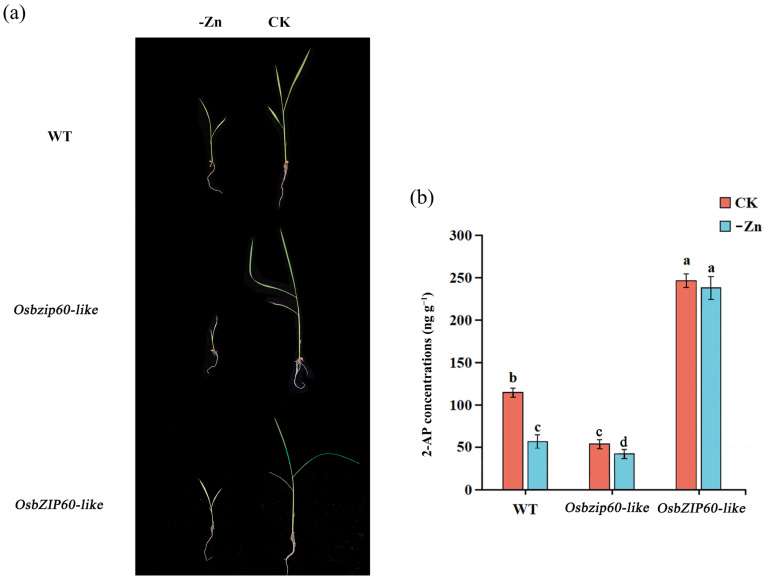
The effect of zinc deficiency on 2-AP accumulation in WT, *Osbzip60*-like mutants and *OsbZIP60*-like over-expressed plants. (**a**) WT, *Osbzip60*-like mutants and *OsbZIP60*-like over-expressed plants were cultured with zinc deficiency for 2 weeks. (**b**) CK: controlled water spray treatment; -Zn: zinc deficiency treatment. Different letters above the bar graphs indicate differences at *p* < 0.05 by the LSD test.

The WT, the *Osbzip60*-like mutants and the *OsbZIP60*-like over-expressing plants were cultured in normal and Zn-deficient medium for two weeks, respectively (Figure 7a). Compared with the controls, the 2-AP concentrations were decreased by 47.82% and 27.27% in the WT and *Osbzip60*-like mutants in Zn-deficient conditions. Moreover, the concentrations of 2-AP in the *OsbZIP60*-like over-expressing plants remained stable after Zn-deficiency treatment (Figure 7b). Overall, the results showed that the *Osbzip60*-like mutant was sensitive to Zn deficiency.

## 3. Discussion

The expression pattern of the *OsP5CS1* gene was consistent with the accumulation pattern of 2-AP content after the foliar Zn application of fragrant rice at the heading stage [3]. In order to further verify that the *OsP5CS1* gene is the node gene in the 2-AP biosynthesis of fragrant rice, CRISPR/Cas9 technology was used to construct the knockout mutant of the *Osp5cs1* gene, and an *OsP5CS1* over-expression vector was constructed to obtain over-expression plants. Compared with the WT, the 2-AP concentrations were significantly decreased in *Osp5cs1* mutants and increased in *OsP5CS1*-over-expressing plants (Figure 2b). The *OsP5CS1* gene was proven to be a key gene in the 2-AP biosynthesis pathway of fragrant rice. There are many studies on the regulation of *P5CS* expression in different plant tissues in response to different environmental signals, but little is known about the transcriptional regulation mechanism of the *P5CS* gene. Cis-acting element analysis was performed on the promoters of rice *OsP5CS1* and *OsP5CS2* genes, respectively. Twenty-four different types of transcription-factor binding sites were predicted in the promoters of these two genes, including the AP2, bZIP, MYB, and NAC families of transcription factors [33]. The transcription factors binding to the promoter of the *OsP5CS1* gene have not been confirmed, and how the expression of the *OsP5CS1* gene is regulated to affect the concentration of 2-AP has not been reported yet. In the present study, yeast one-hybrid assays and EMSA techniques determined that the OsbZIP60-like transcription factor specifically bound to the promoter of the *OsP5CS1* gene (Figure 3 and Figure 4), whereas the OsbZIP60-like was required for the transcriptional activation of *OsP5CS1* gene expression (Figure 5).

**Figure 8:**
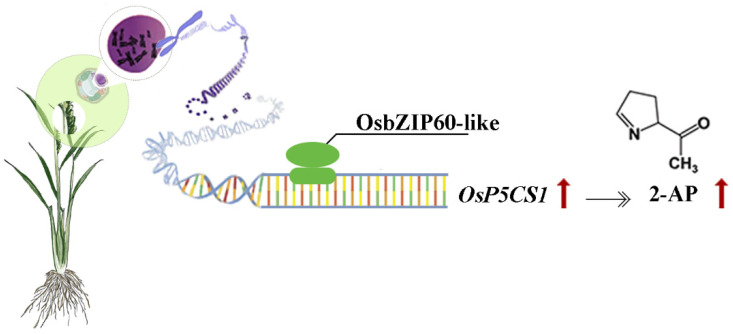
Hypothesized model of OsbZIP60-like regulation of 2-AP content. OsbZIP60-like, as a positive regulator, increased 2-AP content by increasing the expression of the *OsP5CS1* gene. The red arrow indicates up-regulation and the black arrow indicates up-promotion.

Zn-binding proteins account for about 10% of the proteome in eukaryotes [25]. The F-bZIP transcription factors AtbZIP19 and AtbZIP23 were central regulators of the Zn-deficiency response in Arabidopsis, and the double mutants were sensitive to Zn deficiency [31,32]. Under Zn-deficient conditions, AtbZIP19 and AtbZIP23 could bind to the promoter of Zn transport-related genes, activate their transcription, and maintain Zn homeostasis in cells [34]. Under Zn-sufficient conditions, the Zn^2+^ bound to the Cys-His-rich motif of AtbZIP19 and AtbZIP23, which inhibited their own activity and failed to activate the transcription of downstream Zn transport genes (*ZIP* or *NAS*) [35]. OsbZIP48 had the highest sequence similarity with AtbZIP19 and AtbZIP23 in rice and was considered to be a Zn receptor in rice, maintaining Zn homeostasis [30]. However, F-bZIP transcription factors as a Zn receptor for regulating the aroma of fragrant rice have not been reported yet. We used the *OsP5CS1* promoter as bait and screened OsbZIP60-like through yeast one-hybrid assays (Figure 3). Protein domain analysis revealed that OsbZIP60-like belongs to F-bZIP, and amino acids contain Cys and His residues bound to Zn^2+^ (Figure 3d). The EMSA demonstrated that OsbZIP60-like specifically bound to the *OsP5CS1* gene in vitro (Figure 4). The dual luciferase reporting system found that OsbZIP60-like promoted the transcriptional activation of *OsP5CS1* (Figure 5b). Compared with the WT, the *OsP5CS1* gene expression was significantly down-regulated in the *Osbzip60*-like mutant, and the 2-AP concentrations were significantly decreased. The *OsP5CS1* gene expression was significantly up-regulated in *OsbZIP60*-like over-expressed plants, and the 2-AP concentrations were significantly increased (Figure 6c,d). The *Osbzip60*-like mutants were sensitive to Zn-deficient conditions (Figure 7). In this study, OsbZIP60-like acted as a positive regulator to increase 2-AP content by increasing the expression of the *OsP5CS1* gene (Figure 8). However, whether the OsbZIP60-like is a Zn receptor and how OsbZIP60-like as a zinc receptor regulates the mechanism of 2-AP in fragrant rice still need to be explored.

## 4. Materials and Methods

### 4.1. Plant Materials and Experimental Details

The experiment was conducted at the Guangzhou Key Laboratory for Research and Development of Crop Germplasm Resources, Zhongkai University of Agriculture and Engineering, Guangzhou, China (23.104° N, 113.281° E) from January 2021 to June 2022. The *Osp5cs1* knockout mutant and *OsP5CS1* over-expression lines were constructed by the genetic transformation of the Indica rice cultivar, i.e., ‘Zhonghua11’, which knocked out *OsBADH2* to produce fragrance. Three *Osbzip60*-like knockout mutant lines and *OsbZIP60*-like over-expression lines were also constructed with ‘Zhonghua11’, producing aroma as a background material. The wild-type, *Osbzip60*-like mutants, and *OsbZIP60*-like over-expression plants at two weeks of seedling age were cultured in a normal nutrient solution and Zn-deficient medium for two weeks and used to determine the 2-AP concentrations.

### 4.2. Real-Time PCR Quantification of Gene Expression

The RNA was extracted with a MegBio Plant Total RNA Mini-Extraction Kit and then reverse transcribed into cDNA using a Prime Script^®^ RT reagent kit (Shanghai, China) with a gDNA Eraser kit. The relative expression of the *OsP5CS1* gene was analyzed by a Thermo Fisher real-time fluorescence quantitative PCR kit (Waltham, MA, USA). Using the rice actin gene as the endogenous reference gene, three statistical replicates, the primer sequences of *OsP5CS1*, and actin were F 5′-TTTTGAGTCCCGACCTG-3′, R 5′-TTCACCAACATTACGAGGA-3′, F 5′-GATCACTGCCTTGGCTCCTA-3′ and R 5′-GTACTCAGCCTTGGCAATCC-3′, respectively [36].

### 4.3. Determination of the 2-AP Contents

The leaves of 10 g were cut into pieces, and then the 2-AP was extracted by distillation and extraction using a distillation extractor, and a Shimadzu GC-MS QP 2010 plus gas mass spectrometer (Tsujima, Japan) was used. Moreover, 2,4,6-Trimethylpyrimidine (TMP) was used as the internal standard, and the column was a RESTEK Rxi-5ms with a length of 30 cm, an inner diameter of 0.32 mm, and a film thickness of 0.25 μm. The column temperature heating program was as follows: 40 °C for 1 min; then heated to 65 °C at 2 °C min^−1^; held for 1 min; and then heated to 220 °C at 10 °C min^−1^. The carrier gas was high-purity helium (purity > 99.999%), there was constant pressure, and splitless injection mode was employed with 10 μL injection volume. Mass spectrum conditions: electron bombardment (EI) ion source; ion source temperature of 200 °C; the ionization energy was 70 eV; the interface temperature was 250 °C. The temperature of the four-stage rod was 150 °C; we used full scanning mode; and the scanning quality range was *m*/*z* 30–350. The unit was ng g^−1^ [37].

### 4.4. Promoter Cis-Acting Element Analysis

The cis-acting elements of the bZIP60 promoter were analyzed using the Plant CARE website (http://bioinformatics.psb.ugent.be/webtools/plantcare/html/, accessed on 1 January 2022).

### 4.5. Yeast One-Hybrid Assays

The Yeast ‘Y187’ was inoculated in YPDA liquid medium. Three single colonies were randomly selected from the plate, diluted, coated on the corresponding defective plate without adding histidine with different concentrations (0, 10, 20, 30, 40, 50, 75, 100 mM 3AT) of 3AT, and incubated at 30 °C for 3 days. Then, 3AT was a competitive inhibitor of the HIS2 protein, which is a commonly used reporter gene in yeast. In order to reduce the background expression of HIS2 or inhibit self-activation, 3AT at different concentrations was added to the medium. The plasmid containing the *pHIS2:OsP5CS1* promoter was transformed into Y187 yeast as receptive cells, and then plasmid *pGADT7:bZIP60* was transferred into receptive cells and coated on the selected 3AT plate. These positive clones were amplified from yeast cells for DNA sequencing and BLAST analysis against sequences in the GenBank database [30].

### 4.6. Electrophoretic Mobility Shift Assay (EMSA)

The 5′-biotinylated oligonucleotide (5′-ATG CAT GCA TGC ATG CAT GCA TGC-3′) was used as a probe and incubated with the nuclear extract at room temperature for 30 min. The entire reaction mixture was run on a non-denaturing 0.5 × TBE 6% polyacrylamide gel for 1 h at 60 V at 4 °C and then transferred onto Biodyne^®^ B nylon membranes (Pall Corporation, New York, NY, USA). Signals were visualized with reagents included in the kit and ChemiDoc XRS (Bio-Rad Laboratories, Hercules, CA, UAS) [38].

### 4.7. Dual Luciferase Assay System

The *OsP5CS1* promoter (2000 bp) was ligated into the *pGL3:LUC* (containing Firefly luciferase) vector by homologous recombination to construct a reporter gene vector. The *OsbZIP60*-like was connected to a Ubi promoter expression vector. The reporter gene vector, expression vector and *pRT:TK* vector (containing Renilla luciferase) were included in the experimental group, whereas the reporter gene vector, blank control expression vector, and *pRT:TK* vector were included in the control group and were transformed into Arabidopsis protoplasts using the agrobacterium transformation method, respectively. Luciferase and Renilla substrates were added, and the activity of the reporter genes in Firefly luciferase and Renilla luciferase was detected by enzyme marker. The LUC/REN ratio showed the transcriptional regulatory activity of *OsbZIP60*-like on *OsP5CS1* promoters [39].

### 4.8. Statistical Analyses

Data were analyzed by SPSS 25 (Analytical Software, Chicago, IL, USA), whereas the means were separated by the least significant difference (LSD) test at the *p* < 0.05 level.

## 5. Conclusions

The *OsP5CS1* gene was a key gene in the 2-AP biosynthesis pathway of aromatic rice. The OsbZIP60-like transcription factor specifically bound to the *OsP5CS1* gene. The OsbZIP60-like promoted the transcriptional activation of *OsP5CS1*. Compared with the WT, the *OsP5CS1* gene expression was significantly down-regulated in the *Osbzip60-like* mutant, and the 2-AP concentrations were significantly decreased. *OsP5CS1* gene expression was significantly up-regulated in *OsbZIP60*-like over-expressed plants, and the 2-AP concentrations were significantly increased. The *Osbzip60*-like mutants were sensitive to a zinc-deficient environment. Therefore, this study demonstrated that the OsbZIP60-like promoted 2-AP accumulation and might respond to zinc regulation. The relationship between the OsbZIP60-like transcription factor and zinc needs further study.

## Figures and Tables

**Table 1:** Prediction of cis-acting elements in the *OsP5CS1* promoter.

Response Element Type	Number of Cis-Acting Elements
Abscisic acid (ABA) responsiveness	
ABRE	11
ABRE3a	4
ABRE4	4
Salicylic acid (SA) responsiveness	
ERE	1
TCA-element	1
Auxin responsive element	
TGA-element	1
Defense and stress responsiveness	
TC-rich repeats	1
Light responsiveness	
ATCT-motif	4
G-box	10
GATA-motif	1
TCC-motif	2

## Data Availability

Data are contained within the article.
